# Cognitive Training and Transcranial Direct Current Stimulation in Mild Cognitive Impairment: A Randomized Pilot Trial

**DOI:** 10.3389/fnins.2019.00307

**Published:** 2019-04-12

**Authors:** Namrata Das, Jeffrey S. Spence, Sina Aslan, Sven Vanneste, Raksha Mudar, Audette Rackley, Mary Quiceno, Sandra Bond Chapman

**Affiliations:** ^1^Center for BrainHealth, The University of Texas at Dallas, Dallas, TX, United States; ^2^Advance MRI, LLC, Frisco, TX, United States; ^3^Department of Speech and Hearing Science, University of Illinois at Urbana-Champaign, Champaign, IL, United States; ^4^University of North Texas Health Science Center and Department of Internal Medicine and Geriatrics and TCU/UNTHSC, School of Medicine, Department of Medical Education, Fort Worth, TX, United States

**Keywords:** mild cognitive impairment, Alzheimer’s disease, transcranial direct current stimulation, cerebral blood flow, fMRI, cognitive training, strategic memory advanced reasoning training, brain modulation

## Abstract

**Background:**

Transcranial direct current stimulation (tDCS), a non-invasive stimulation, represents a potential intervention to enhance cognition across clinical populations including Alzheimer’s disease and mild cognitive impairment (MCI). This randomized clinical trial in MCI investigated the effects of anodal tDCS (a-tDCS) delivered to left inferior frontal gyrus (IFG) combined with gist-reasoning training (SMART) versus sham tDCS (s-tDCS) plus SMART on measures of cognitive and neural changes in resting cerebral blood flow (rCBF). We were also interested in SMART effects on cognitive performance regardless of the tDCS group.

**Methods:**

Twenty-two MCI participants, who completed the baseline cognitive assessment (T1), were randomized into one of two groups: a-tDCS + SMART and s-tDCS + SMART. Of which, 20 participants completed resting pCASL MRI scan to measure rCBF. Eight SMART sessions were administered over 4 weeks with a-tDCS or s-tDCS stimulation for 20 min before each session. Participants were assessed immediately (T2) and 3-months after training (T3).

**Results:**

Significant group × time interactions showed cognitive gains at T2 in executive function (EF) measure of inhibition [DKEFS- Color word (*p* = 0.047)], innovation [TOSL (*p* = 0.01)] and on episodic memory [TOSL (*p* = 0.048)] in s-tDCS + SMART but not in a-tDCS + SMART group. Nonetheless, the gains did not persist for 3 months (T3) after the training. A voxel-based analysis showed significant increase in regional rCBF in the right middle frontal cortex (MFC) (cluster-wise *p* = 0.05, *k* = 1,168 mm^3^) in a-tDCS + SMART compared to s-tDCS + SMART. No significant relationship was observed between the increased CBF with cognition. Irrespective of group, the combined MCI showed gains at T2 in EF of conceptual reasoning [DKEFS card sort (*p* = 0.033)] and category fluency [COWAT (*p* = 0.055)], along with gains at T3 in EF of verbal fluency [COWAT (*p* = 0.009)].

**Conclusion:**

One intriguing finding is a-tDCS to left IFG plus SMART increased blood flow to right MFC, however, the stimulation seemingly blocked cognitive benefits of SMART on EF (inhibition and innovation) and episodic memory compared to s-tDCS + SMART group. Although the sample size is small, this paper contributes to growing evidence that cognitive training provides a way to significantly enhance cognitive performance in adults showing memory loss, where the role of a-tDCS in augmenting these effects need further study.

## Introduction

Mild cognitive impairment (MCI) is a stage in which individuals endorse subtle changes in cognitive functions that are corroborated on objective assessments of cognition but have minimal changes in functional abilities (Weiner et al., 2013; Cohen and Klunk, 2014). The rate of conversion from MCI to a diagnosis of Alzheimer’s disease (AD) is around 10–15% per year (Manly et al., 2008; Roberts et al., 2014). To date, pharmacological interventions have failed to show realizable benefits in mitigating cognitive decline in individuals with MCI and in preventing progression to AD (Andrieu et al., 2015). As a result, there is growing interest in exploring the benefits of non-pharmacological interventions such as lifestyle modifications (nutrition and exercise) (Morris, 2009; Erickson et al., 2011), cognitive training (Jean et al., 2010; Belleville et al., 2011; Chapman and Mudar, 2014; Chapman et al., 2017; Edward et al., 2017; Harvey et al., 2018), and repetitive non-invasive brain stimulation such as Transcranial Magnetic Stimulation (rTMS) and Transcranial Direct Current Stimulation (tDCS) (Flöel et al., 2012; Elder and Taylor, 2014).

Decades of research on the effectiveness of process-based and strategy-based cognitive training have shown that training protocols that target higher-order cognitive functions (e.g., reasoning) and are strategy-based yield broad cognitive benefits across clinical groups and in individuals with MCI, in particular (Edward et al., 2017). For instance, our group has shown that strategic memory and advanced reasoning training (SMART), previously referred to as gist reasoning training, improves top–down cognitive processes and associated training-related neural outcomes Specifically, benefits of SMART gains have been reported as increased executive functions and enhanced neural functions in cognitively normal older adults (Anand et al., 2011; Chapman et al., 2015, 2017; Motes et al., 2018) and in adults with traumatic brain injury (Vas et al., 2011, 2015; Cook et al., 2014; Han et al., 2017). Consistent findings of increased resting cerebral blood flow (rCBF) to specific areas of the brain were associated with cognitive gains following SMART in cognitively normal older adults (Chapman et al., 2016), adults with TBI (Vas et al., 2015), and adults with bipolar disorder (Venza et al., 2016). In our previous study with SMART training in healthy aging, we demonstrated increases in global and regional blood flow in bilateral medial orbital frontal cortex (mOFC), a part of inferior fontal gyrus (IFG), and posterior cingulate cortex (PCC) and associated cognitive gains (Chapman et al., 2015, 2017). The IFG is of particular interest because it purportedly supports a cognitive control network of complex mental processes associated with executive functions including reasoning, working memory, and inhibition of unwanted information required for goal-directed behavior (Rubia et al., 2003; Aron et al., 2004). In AD animal models, IFG is shown to aid in the maintenance of the cognitive performance, whereas in adults with genetic risk of AD, IFG seems to support the compensatory mechanism in the brain (Wishart et al., 2006; Filbey et al., 2010). Furthermore, functional imaging studies have associated greater BOLD activity of IFG to better cognitive outcomes (Diamond et al., 2007) and memory recovery success in AD patients (Grady, 2012).

Gist reasoning training has also shown to be beneficial in individuals with MCI (Mudar et al., 2017, 2019). In a separate but recently completed randomized pilot study, MCI individuals who underwent SMART improved in strategic processing and attention during a list learning task and on a concept abstraction measure relative to an active control group that received new learning of relevant facts about brain health (Mudar et al., 2017). Not only did the SMART trained group show significant improvement on cognitive and self-reported memory measures, but training-related modulations in neural functions were also noted. With regard to neural changes, MCI individuals who underwent SMART training showed enhanced event-related desynchronization in low-frequency alpha band (8–10 Hz) on response inhibition (NoGo) trials and high-frequency alpha band (11–13 Hz) on response execution (Go) trials relative to the active control group (Mudar et al., 2019).

Given the growing evidence of both cognitive and neural benefits of reasoning training (SMART), the next logical question to examine was whether benefits of SMART for individuals with MCI can be augmented using brain stimulation approaches such as tDCS when combined with cognitive training. tDCS is a non-invasive brain stimulation approach used to modulate cortical functioning by applying weak direct current over the scalp (Nitsche et al., 2007). Recent studies have begun to explore the cognitive benefits of tDCS alone in MCI (Biundo et al., 2015; Manenti et al., 2016; Yun et al., 2016). In a randomized clinical trial involving 16 individuals with MCI, Yun et al. (2016) investigated if anodal direct current stimulation (a-tDCS) over left dorsolateral prefrontal cortex (DLPFC) with reference electrode over right DLPFC for 30 min over nine sessions in 3 weeks could enhance memory. Compared to the sham group, significant improvement was observed on a Multifactorial Memory Questionnaire (MMQ) with questions probing on how individual feel about their memory and mistakes. Similarly, Murugaraja et al. (2017) findings in 10 individuals with MCI demonstrated that 20 min of 2 mA anodal stimulation over the left DLPFC with a reference electrode on right supraorbital region for five consecutive sessions significantly improved immediate and delayed recall of pictures over an extended period of 1 month. A study by Meinzer et al. (2015) examined the effect of a-tDCS on brain function in individuals with MCI on semantic word-retrieval using fMRI. A 1 mA intensity over 20 min applied over left IFG showed improvement in semantic word retrieval task with a decrease in task-related prefrontal hyperactivity supporting enhanced processing efficacy.

The body of research supporting the cognitive benefits of a-tDCS in MCI, used alone, is growing; however, no study to our knowledge has yet examined the combined effects of tDCS and cognitive training in MCI. A study by Cotelli et al. (2014) in patients with mild to moderate AD provides support to motivate the present study. Their team examined the effects of combined tDCS applied to the DLPFC and individualized computerized (IC) memory training on memory improvements. Their findings of significant improvement in face-name association memory task suggest that there may be a value in exploring such combined therapies in individuals at earlier stages of dementia, specifically those with MCI.

The goals of this study were three-fold. First, we investigated whether anodal tDCS to left inferior frontal gyrus (IFG) combined with SMART training (a-tDCS + SMART) would show significant cognitive gains over an extended period (i.e., 3 months post-training) compared to the sham tDCS and SMART training group (s-tDCS + SMART). Based on evidence summarized above showing neural gains after SMART training in MCI and healthy controls combined with evidence for IFG vulnerability in AD, we chose to stimulate the region over the left IFG. We hypothesized that a-tDCS to left IFG delivered for 20 min just prior to participating in SMART training would enhance the cognitive benefits. The potential to enhance cognitive-training benefits with neuromodulation is based on a hypothesis that the brain’s inherent neuroplasticity can be ‘primed’ to be more responsive to intervention protocols (Meinzer et al., 2015; Reinhart et al., 2017). Secondly, we examined whether a-tDCS + SMART significantly altered rCBF, a measure of neural function previously identified in clinical training trials involving other clinical populations, relative to s-tDCS + SMART. We focused on rCBF to measure neural health based on a series of cognitive training trials where fMRI findings revealed that rCBF was an early and sensitive measures of improved cognitive brain health following interventions (Chapman et al., 2015, 2016, Vas et al., 2016; Venza et al., 2016). We expected that the a-tDCS + SMART would bring about greater changes to rCBF as compared to the s-tDCS + SMART, given the enhanced potential to harness neural plasticity shown by previous tDCS trials (Yun et al., 2016). Finally, we wanted to explore whether SMART training improved cognitive functions irrespective of the a-tDCS or s-tDCS group. Based on prior results showing adults with MCI benefitted from SMART protocol (Mudar et al., 2017, 2019), we proposed that both groups would show cognitive gains.

## Materials and Methods

This blinded randomized clinical trial (ClinicalTrials.gov ID: NCT02588209) study using tDCS in individuals with MCI was approved by the Institutional Review Boards (IRB) of The University of Texas Southwestern Medical Center (UTSW IRB STU082015-031) and The University of Texas at Dallas (UTD IRB# 15-97). All eligible participants signed informed consent under the guidelines of UTSW and UTD IRBs under the Declaration of Helsinki 1975 revised in 1981.

### Participants

Twenty-two (22) participants with a diagnosis of MCI based on either Petersen’s (Petersen et al., 2001) or Alzheimer’s disease neuroimaging initiative (ADNI, Aisen et al., 2010) criteria were included in the study from the Dallas Fort Worth community. The diagnosis of MCI was confirmed by the research team consisting of a neurologist, cognitive neuroscientist, and speech-language pathologist. The comprehensive Petersen’s or ADNI criteria used for enrollment were: (1) subjective memory complaints; (2) objective memory loss measured by either logical memory subtest from Wechsler Memory Scale-III (WMS-III, Wechsler, 1997b) with delayed memory recall scores of 9–11 for 16 or more years of education, 5–9 for 8–15 years of educational and 3–6 for 0–7 years of education or California Verbal Learning Task (CVLT) delayed memory recall of -1.5 standard deviation below the mean; (3) preserved daily functional activities; (4) clinical dementia rating scale of 0.5 (CDR, Morris, 1993); (5) MMSE of 24–30 (Folstein et al., 1975); and (6) without symptoms of dementia. Subjective memory concerns of each participant were assessed using a Multifactorial Memory Questionnaire (MMQ, Troyer and Rich, 2002). The questionnaire included 57 items divided into three subscales: MMQ-Contentment (MMQ-C), MMQ-Ability (MMQ-A), and MMQ-Strategies (MMQ-S). MMQ-C assessed participants’ self-satisfaction and concerns of their memory in which higher scores indicated greater satisfaction with one’s memory. MMQ-A measured self-perception of memory ability with higher scores indicating better self-reported memory ability. Whereas, MMQ-S assessed individuals use of memory strategies in daily life with higher scores reflecting greater use of memory strategies. Irrespective of sex and ethnic groups other inclusion factors were right-handed individuals, age 50–80 years with a minimum of 12 years’ education. All participants were assessed for signs of depression using the geriatric depression scale (GDS, Yesavage et al., 1982) and only participant with no or mild depression were in the study (see Table 1). The ability to read at the level of 12th grade was assessed using the Wide Range Achievement Test (WRAT4, Wilkinson and Robertson, 2006).

**Table 1:** Demographics and clinical characteristics (Cognitive Screening Measures).

Measures	Total group (Mean ±*SD*)	Anodal tDCS group (Mean ±*SD*)	Sham tDCS group (Mean ±*SD*)	*p*-values
*Demographics*				
Number	22	12	10	
Age	62.91 ± 7.79	62.58 ± 8.43	63.30+7.38	0.836
Education	17.14 ± 3.20	17.92 ± 3.94	16.20 ± 1.75	0.218
Sex (Females: Males)	15:7	8:4	8:2	0.300
*Cognitive screening measures*				
CDR	0.5	0.5	0.5	
MMSE	27.91 ± 1.34	28 ± 0.95	27.80 ± 1.75	0.737
GDS	2.05 ± 1.70	2 ± 1.65	2 ± 1.85	0.895
LM Immediate Recall	11.41 ± 2.64	11.17 ± 2.17	11.70 ± 3.20	0.647
LM Delayed Recall	10.36 ± 2.34	10.42 ± 2.11	10.30 ± 2.71	0.911
CVLT Immediate Recall	8.23 ± 3.56	6.75 ± 3.05	10 ± 3.43	0.029^*^
CVLT Delayed Recall	9.14 ± 3.33	7.83 ± 3.38	10.70 ± 2.63	0.041^*^


*CDR, Clinical Dementia Rating Scale; MMSE, Mini Mental Status Examination; GDS, Geriatric Depression Scale; LM, Logical Memory; CVLT, California Verbal Learning Task. There was no difference between the participants’ demographics, *p* > 0.05. ^∗^Indicates significant at *p* < 0.05.*

Exclusion criteria of the study were: less than 12th grade of education, left-handed, unable to speak, read, and write English; CDR value of 0 or >0.5, previous or present diagnosis of neurological disorders such as stroke, brain tumor, cerebral hemorrhage; autoimmune diseases such as fibromyalgia, systemic lupus erythematosus (SLE), multiple sclerosis (MS) and rheumatoid arthritis (RA); uncontrolled metabolic disturbances such as uncontrolled diabetes mellitus, thyroid disorders; psychiatric disorders like bipolar disorder, major depressive disorder, pervasive developmental disorder, schizophrenia, and anxiety disorder; history of substance abuse; head injuries, cancer patients with a history of radiation or chemotherapy. Participants with metallic objects, permanent makeup, medical devices in the body were excluded from the study. Moreover, participants on antidepressants, sedatives, anxiolytics, neuroleptics medications were also denied to take part in the study as it interfered with tDCS stimulation.

**FIGURE 1:**
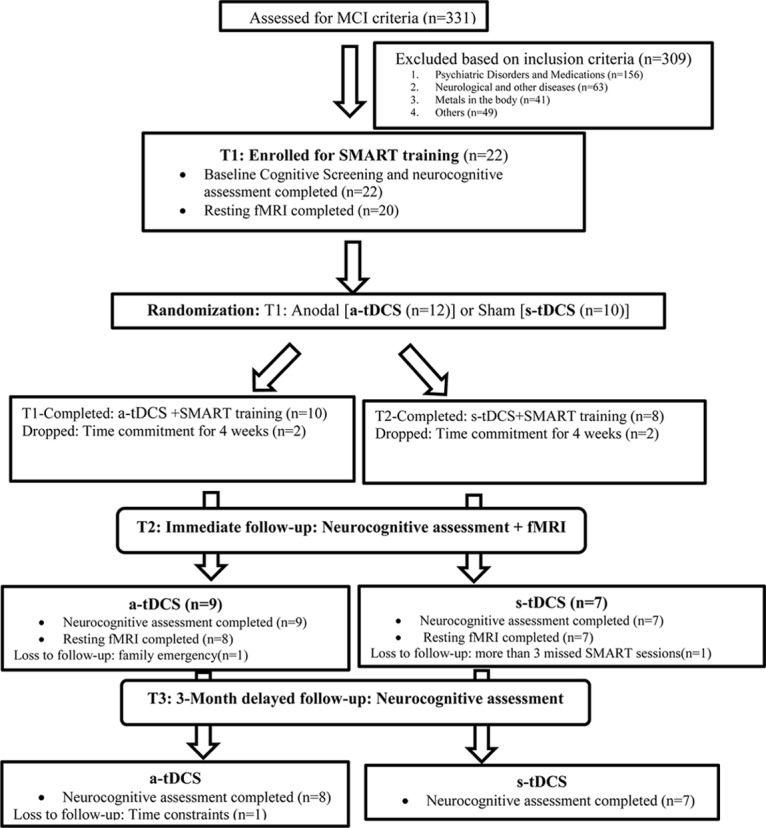
The flow chart of participants follow-up through the research.

All 22 participants completed baseline neurocognitive assessments of which 20 completed resting state pCASL MRI before training (T1). A research assistant, who was blinded to all the participant information and cognitive behavior, randomized the participants into one of two groups: either the a-tDCS or the s-tDCS group following by SMART training using random function on Microsoft excel sheet, after the initial baseline assessment. The research assistant was not involved in either the neurocognitive assessments or trainings. Post-SMART training (T2), 16 subjects completed the neurocognitive assessment of which 15 participants had the follow-up scan. Finally, at 3-month post-training (T3) follow-up, 15 individuals were assessed for neurocognitive behavior only. The complete breakdown of the participant enrollment and follow-up is summarized in Figure 1.

### Neurocognitive Measures

A 3-h neurocognitive test battery was administered on a non-training day at three time points i.e., before training (T1), post-training (T2), and 3-month post training (T3). The measures included the cognitive domains of executive function, and memory summarized in Table 2. Twenty-two (22) participants completed the baseline cognitive assessment and sixteen (16) immediate post-training assessment. Fifteen (15) completed 3-month follow-up assessment. For details on the follow-up sessions see Figure 1.

**Table 2:** Neurocognitive measures, memory questionnaire and memory screening measures administered to the participants.

Cognitive Domain	Measures	Description
**Executive Function**
(1) Complex abstraction	Test of Strategic Learning (TOSL) (Chapman et al., 2002)	Examined the ability to condense and synthesize lengthy information written as a summary from a complex text. Scores represents number of abstracted ideas.
(2) Innovation	Test of Strategic Learning (TOSL) (Chapman et al., 2002)	Assessed the ability to construct as many interpretations as possible from the same text above. Scores represent fluency of abstracted idea generation.
(3) Fluency: Verbal/Category	Controlled Oral Word Association (COWAT) (Benton et al., 1994; Spreen and Strauss, 1998)	Examined the ability to generate as many words staring with a particular alphabet or a category in 1 min.
(4) Inhibition	Delis–Kaplan executive function system (DKEFS) color word interference test (Delis et al., 2001)	Examined the ability to inhibit from reading color of printed word instead of reading the word. Scored as time taken to complete the task.
(5) Conceptual Reasoning	Delis–Kaplan executive function system (DKEFS) card sort (Delis et al., 2001)	Examined the ability to draw similarities between two sets of cards by drawing reasons behind the selection of cards.
**Memory**
(1) Episodic Memory: Memory for facts	Test of Strategic Learning (TOSL) (Chapman et al., 2002)	Examined the ability to recall details of a complex short story.
(2) Complex Memory	Selective Auditory learning task (Hanten et al., 2007)	Examined the ability to focus and pay attention to high-priority stimulus, while simultaneously blocking or inhibiting unwanted or low-priority information.
**Subjective memory perception**
(1) Memory questionnaire	Multifactorial Memory Questions (MMQ) (Troyer and Rich, 2002)	Examined the individual’s self-perception of memory in three subscales using 57 items questionnaire (1) MMQ-Contentment (MMQ-C): Self-satisfaction of memory (2) MMQ-Ability (MMQ-A): Self-perception of memory (3) MMQ-Strategy (MMQ-S): Using of memory strategies in daily life functions.
**Screening Memory measures**	(1) California Verbal Learning Task (Petersen et al., 2001)	Examined the ability to recall a list of sixteen (16) words in four categories immediately after the list was read followed by delayed recall after 20 min interval.
	(2) Logical Memory (ADNI Criteria, WMS-III, Wechsler, 1997b)	Examined the ability to recall a short story as it is read out immediately and after 20 min interval.


### tDCS Stimulation

Direct current was provided through a battery-driven stimulator (DC-Stimulator Plus, neuroConn GmbH). To insure consistency of electrode placement on each participant’s skull over the left IFG brain region, the placement of the anodal electrode was calculated as 0.5 cm above the left eyebrow and 1cm on the left forehead away from the center of the nasal bridge. Research assistants were trained to maintain consistency of IFG area stimulation across all the participants. The localization of IFG was based on prior research wherein anodal electrode positions were defined according to the 10–20 EEG system (Iyer et al., 2005; Cattaneo et al., 2011; Meinzer et al., 2015). In the EEG simulating studies IFG anodal electrode was positioned at the center of line connecting between (a) and (b) wherein (a) the intersection of T3-F3 and F7-C3 and (b) is the midpoint between F7-F3 on the left side of the head. The stimulating electrode was inserted in a 3 × 5 cm^2^ saline-soaked synthetic sponge, and was centered FG as previous studies found significantly improved semantic fluency with this electrode montage over IFG (Iyer et al., 2005; Cattaneo et al., 2011; Meinzer et al., 2015). The reference electrode (3 × 5 cm^2^) was positioned over the contralateral shoulder. tDCS was delivered with a constant current of 2mA during resting-state. The a-tDCS group received the stimulation for 20 min (10 s fade-in and 10 s fade-out). The s-tDCS received stimulation for a total of 20 s (10 s fade-in and 10 s fade-out) to mimic the sensation of the stimulation. As the SMART training occurred in groups of 2–5 participants irrespective of the group assignment (a-tDCS versus s-tDCS) all individuals were attached to the machine with the electrode for a total of 20 min irrespective of the group assignment. Each SMART training session (total of 8 sessions spread over 4 weeks) was coupled with tDCS session just before the training. To maintain some consistency of mental processing during the tDCS sessions prior to training, everyone watched Planet Earth videos.

### SMART: Cognitive Training Protocol

In previous MCI research, SMART training was labeled as gist reasoning training (Mudar et al., 2017, 2019). Detailed information about the specifics of the training can be found in Mudar et al. (2017, 2019). Training was delivered to all participants in both groups in sessions involving small groups of 2–5 individuals over 4 weeks, consisting of two 1-h sessions per week for a total of 8 h of training. The training is strategy-based rather than content-based so that the focus is not content specific or situation dependent, but hierarchical with each strategy building upon previous strategies to transform the concrete meaning into abstracted gist-based meanings through reasoning and inferencing. Participants received a-tDCS or s-tDCS stimulation immediately prior to each of the SMART training sessions.

### MRI Experiment

MRI scans were completed using 3-Tesla (Philips Medical System, Best, The Netherlands within one (1) week before (T1) and after SMART training (T2) but not at 3-month training (T3). A body coil was used for radiofrequency (RF) transmission and a 32-channel head coil with parallel imaging capability was used for signal reception. We used a pCASL (pseudo-Continuous Arterial Spin Labeling) sequence to measure cerebral blood flow (CBF) at rest. Additionally, a high-resolution *T*_1_-weighted image was acquired as an anatomical reference. The details of imaging parameters and their processing techniques are provided below.

Imaging parameters for pCASL experiments were: single-shot gradient-echo EPI, field-of-view (FOV) = 240 × 240, matrix = 80 × 80, voxel size = 3 mm × 3 mm, 29 slices acquired in ascending order, slice thickness = 5 mm, no gap between slices, labeling duration = 1650 ms, post-labeling delay = 1525 ms, time interval between consecutive slice acquisitions = 35.5 ms, TR/TE = 4260/14 ms, SENSE factor 2.5, number of controls/labels = 45 pairs, RF duration = 0.5 ms, pause between RF pulses = 0.5 ms, labeling pulse flip angle = 90°, bandwidth = 2.7 kHz, echo train length = 35, and scan duration 6.5 min. The high-resolution *T*_1_-weighted image parameters were magnetization prepared rapid acquisition of gradient-echo (MPRAGE) sequence, TR/TE = 8.3/3.8 ms, shot interval = 2100 ms, inversion time = 1100 ms, flip angle = 12°, 160 sagittal slices, voxel size = 1 mm × 1 mm × 1 mm, FOV = 256 mm × 256 mm × 160 mm, and duration 4 min.

pCASL image series were realigned to the first volume for motion correction (SPM8’s realign function, University College London, United Kingdom). An in-house MATLAB (Mathworks, Natick, MA, United States) program was used to calculate the difference between averaged control and label images. Then, the difference image was corrected for imaging slice delay time to yield CBF-weight image, which was normalized to the Brain template from Montreal Neurological Institute (MNI). Last, the absolute CBF was estimated by using Alsop and Detre’s equation in the units of mL blood/min/100 g of brain tissue (Aslan et al., 2010).

### Statistical Analyses

For the behavioral outcomes, we modeled each respective dependent variable as a constrained linear mixed effects modely_ijk_ = μ_0_ + δ_ij_ + ε_ijk_, where μ_0_ is the common baseline mean prior to randomization into *i* = 1, 2 groups (a-tDCS + SMART or s-tDCS + SMART); δ_ij_is mean change from baseline by *j* = 1,2 time periods (immediate (T2) or 3-month delay (T3)post-training); and ε_ijk_ are random errors, which are independent across the k = 1,...n_i_ subjects but positively correlated across the j = 0, 1, 2 time. We applied the Benjamini-Hochberg method (Benjamini and Hochberg, 1995) to control the false discovery rate (FDR) over the multiple tests.

For voxel-based analyses (VBA), the individual CBF maps were spatially smoothed with full-width half-maximum kernel of 6 mm, following the pre-processing of the images. Mixed linear effects models, as described above, were then applied to each voxel’s rCBF measure, except for the fact that *j* = 0, 2 only (i.e., no T3 images were obtained). To control for multiple testing across voxels, we employed standard cluster extent inference, using the function *3dClustsim* (with –acf option) in AFNI (NIMH Scientific and Statistical Computing Core, Bethesda, MD, United States) and a voxel-level threshold of 0.005.

Our hypotheses concerned the two interaction effects from the mixed model, as well as the two time effects from the mixed model. Specifically, to test the effects of tDCS on both rCBF and behavioral measures, we derived *t*-statistics from the two interaction contrasts δ_1j_ - δ_2j_ for j = 1,2, corresponding to group differences between immediate- or 3-month-delay post-training mean changes from baseline, respectively To test the “main effect” of time (i.e., effects due to cognitive training, in the absence of an interaction) we derived t-statistics from the contrasts (δ_1j_ + δ_2j_)/2, the mean change from baseline, averaged over the two treatment groups for each of the post-training time periods.

Finally, at each voxel, we modeled change in rCBF as group-specific regressions on changes in behavioral measures to test for neural/behavioral relationships. Specifically, we used the linear model Δz_ik_ = β_0i_ + β_1i_ ⋅ Δy_ik_+ ∈_ik_ and derived t-statistics from the contrasts β_11_ - β_12_ to test whether the relationship depended on the tDCS treatment or from β_11_ - β_12_/2 to test whether the relationship was common to both treatment groups.

## Results

### Effect of tDCS on SMART

**FIGURE 2:**
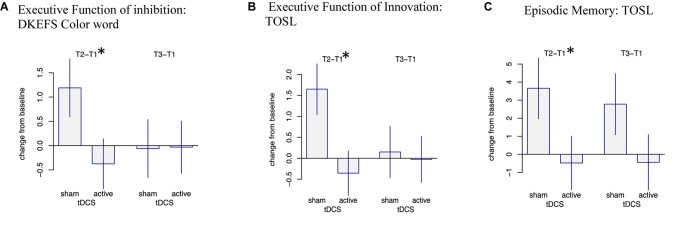
Mixed Model Effects: Immediate cognitive gains (T2-T1) in s-tDCS + SMART group **(A)** Executive Function of Inhibition: DKEFS Color word interference (*p* = 0.047), **(B)** Executive Function of Innovation: TOSL (*p* = 0.010), and **(C)** Episodic Memory: Test of Strategic Learning (TOSL) (*p* = 0.048) relative to the a-tDCS + SMART group. ^∗^Indicates significant change (T2-T1) at *p* < 0.05.

Overall, we observed three significant two-way interactions between groups and training effect over time. The s-tDCS + SMART group showed significant immediate cognitive gains (T2 to T1) in executive functions of inhibition [DKEFS-Color word interference (*t* = -2.04, *p* = 0.047)] and innovation [TOSL (*t* = -2.67, *p* = 0.010)], and episodic memory as measured by retrieval of facts from a lengthy text [TOSL (*t* = -2.03, *p* = 0.048)]; whereas the a-tDCS + SMART training showed no such gains (Table 4 and Figure 2). Nonetheless, the cognitive benefits did not last for over 3-months after training (T3 to T1).

### Effect of tDCS on CBF

**FIGURE 3:**
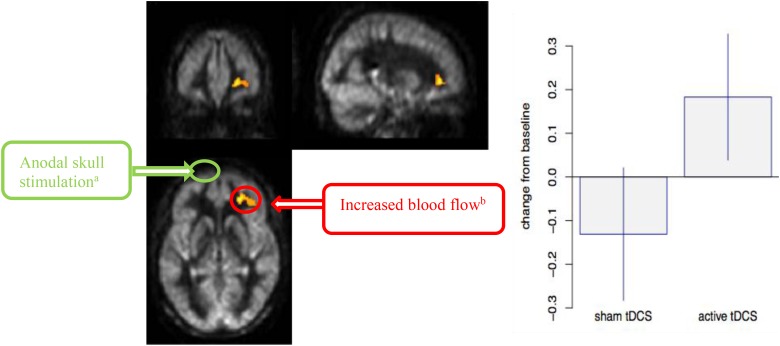
Cerebral blood flow (CBF) voxel-based analysis for the interaction contrast, superimposed on average CBF map of all participants. Right Middle Frontal Cortex (MFC) was significant at a cluster *p*-value = 0.05 (*k* = 1,168 mm^3^). Representation of the anodal stimulation site (green circle) and increased CBF. We illustrate the contralateral nature of the anodal stimulation from the CBF changes. **(A)** Anodal skull stimulation over *left* inferior frontal gyrus (IFG) for a total 8 sessions for 20 min prior to cognitive training over 4-week period. **(B)** Increased blood flow in *right* MFC after completing cognitive training sessions(T2-T1).

Figure 3 shows the interaction of VBA results between a-tDCS + SMART group versus s-tDCS + SMART group, testing the increase from T1 to T2 due to tDCS stimulation. A significantly larger increase in blood flow was observed at the right middle frontal cortex (MFC) in the a-tDCS + SMART compared to the s-tDCS + SMART, cluster-wise at *p* = 0.05, *k* = 1,168 mm^3^. Table 3 summarizes these findings for cluster-level inference as well as descriptive statistics for peak voxel within cluster. We did not find significant relationships between neurocognitive changes and rCBF changes between a-tDCS + SMART and s-tDCS + SMART groups.

**Table 3:** Brain regions that showed significant cerebral blood flow (CBF) increase at rest in Active group compared to SHAM group.

			*MNI*			
				
Brain Regions	BA	Cluster Size (mm^3^)	*X*	*Y*	*Z*	*T*-Value
*Sham < Active*						
Right Middle Frontal Cortex	10	1,168	24	44	-2	6.1


*The coordinates depict the peak of clusters.*

**Table 4:** Mixed Model: Interaction effects [Group (a-tDCS versus s-tDCS) × Time effect (SMART training)]: Immediate cognitive gains (T2-T1), and Delayed cognitive gains (T3-T1) changes in cognitive function in mild cognitive impairment (MCI).

Screening and Neurocognitive Measures	Immediate cognitiv gains (T2-T1)		Delayed cognitive gains (T3-T1)	
		
	*t*-statistics	*p*-value	*t*-statistics	*p*-value
**Executive Functions**				
(1) Inhibition				
(a*) DKEFS-Colorword interference (Switching and Inhibition)*	-2.04	0.047^∗^	0.04	0.971
(2) Complex abstraction				
(a*) TOSL*	-0.27	0.79	-1.01	0.315
(3) Innovation				
(a*) TOSL*	-2.67	0.010^∗^	-0.23	0.820
**Memory**				
(1) *Episodic Memory: Memory for facts*				
*(a) TOSL*	-2.03	0.048^∗^	-1.55	0.127


*DKEFS, Delis–Kaplan Executive Function System^*TM*^, TOSL, Test of Strategic Learning. *p*-values < 0.05^∗^(significant) specified tests of interaction contrasts.*

### Effect of Cognitive Training Averaged Over Groups

When we averaged both groups (a-tDCS and s-tDCS), we observed significant immediate cognitive improvement (T2 to T1) in executive functions of conceptual reasoning [DKEFS card sort (*p* = 0.033)] and category fluency (*p* = 0.055) along with later-emerging cognitive gains (T3 to T1) of verbal fluency (*p* = 0.009). Additionally, we showed immediate (T2 to T1) and persisted gains (T3 toT1) in self-evaluation of memory contentment and satisfaction(MMQ-C) e.g., confidence in remembering things [T2 to T1: MMQ-C (*p* = 0.003) and T3 to T1: MMQ-C (*p* = 0.000)], ability to make less mistakes on memory task (MMQ-A) e.g.*, paying bills on time* [T2 to T1: MMQ-A *(p* = 0.000) and T3to T1: MMQ-A (*p* = 0.002)] with significant improvement in applying memory strategies, e.g., organizing information one wants to remember at 3-month post training[T3 to T1: MMQ-S (*p* = 0.044)]. Finally, we observed improvement on an objective memory measure (CVLT), used also for screening purposes, immediately after training (T2 to T1) in immediate recall (*p* = 0.002) and delayed recall of words (*p* = 0.001) with the gains maintained after 3 months training (T3 to T1) in both immediate recall (*p* < 0.001), and delayed recall (*p* = 0.020) of words (Table 5).

**Table 5:** Mixed Model: Time Effect on cognitive measures irrespective of group assignment: Immediate cognitive gains (T2-T1), and Delayed cognitive gains (T3-T1) changes in cognitive function in mild cognitive impairment (MCI).

Neurocognitive Measures	Immediate cognitive gains (T2-T1)		Delayed cognitive gains (T3-T1)	
		
	*t*-statistics	*p*-value	*t*-statistics	*p*-value
**Executive Functions**				
(1) Conceptual Reasoning				
*(a) DKEFS Card Sort*	2.22	0.032^*^	1.54	0.015^*^
(2) Fluency				
*(a) Verbal Fluency (COWAT)*	0.88	0.385	2.82	0.009^**^
*(b) Category Fluency (COWAT)*	1.99	0.055^*^	1.38	0.176
3. Complex abstraction	0.96	0.343	0.89	0.379
**Memory**				
(1) Complex Memory				
(a) *Selective Auditory Learning* Task				
(i) Trial 1	-0.27	0.294	-0.50	0.620
(ii) Trial 2	0.56	0.580	1.68	0.101
(iii) Trial 3	0.49	0.624	1.07	0.292
**Subjective Memory Evaluation (MMQ)**				
*(a) MMQ Contentment*	3.29	0.003^**^	3.92	<0.001^**^
*(b) MMQ Ability*	5.17	<0.001^**^	3.39	0.002^**^
*(c) MMQ Strategies*	1.07	0.294	2.10	0.044^*^
**Memory Screening Measures (CVLT)**				
*(a) CVLT: Immediate Recall*	3.40	0.002^**^	4.22	0.000^**^
*(b) CVLT: Delayed Recall*	3.63	0.001^**^	2.43	0.020^*^


*DKEFS, Delis–Kaplan Executive Function System^*TM*^; MMQ, Multifactorial Memory Questionnaire; COWAT, Controlled Oral Word Association Test; CVLT, California Verbal Learning Task. ^∗^*p*-values refers to the mixed model effects. ^∗∗^Significance at 5% false discovery rate (FDR) over the multiple tests.*

## Discussion

The present randomized pilot trial represents a concerted effort to explore the potential benefits of a complementary non-pharmacological treatment strategy of adding transcranial direct current stimulation prior to cognitive training sessions in MCI, a population at risk population for Alzheimer’s disease. Our goal was to determine whether a-tDCS + SMART combined intervention protocol would work synergistically to increase gains above any benefits from a cognitive training protocol (i.e., SMART) alone, previously shown to benefit MCI in separate studies (Chapman and Mudar, 2014; Mudar et al., 2017). We examined post-intervention effects in cognitive abilities and neuronal health immediately after training (T2) and whether any gains would be maintained at 3 months post-training (T3) to motivate a larger trial. The study examined differences in neuronal health using measures of rCBF to better understand the neural mechanisms underlying changes resulting from a-tDCS + SMART versus s-tDCS + SMART. Additionally, the study examined the effects of cognitive training immediately (T2) and 3 months (T3) after training when both tDCS groups (a-tDCS + SMART, s-tDCS + SMART) were combined as single group for analyses.

Three findings emerged from this randomized pilot study. The primary hypothesis was that a-tDCS, delivered to the left frontal brain region for 20 min prior to each training session, would incrementally improve the effects of cognitive training over training alone (s-tDCS). This prediction was not supported. Instead, the group with a-tDCS stimulation to the left inferior frontal gyrus (IFG) prior to each training session failed to show significant gains on select measures of interest (i.e., inhibition, innovation and memory for facts) after training compared to the s-tDCS + SMART group at T2 or T3. In contrast, the s-tDCS + SMART group showed significant immediate gains (T2) on two measures of executive function, both inhibition and innovation, and on a memory for facts (episodic memory) measure as compared to the a-tDCS group. Second, our analyses of regional resting cerebral blood flow (rCBF) revealed a significant immediate increase in the right middle frontal cortex (MFC) (T2-T1) in the a-tDCS + SMART group when compared to the sham + SMART group. These two findings of between-group differences taken together, suggest that the a-tDCS did indeed modulate cognitive and neural plasticity, just not in the expected manner. The findings suggested that the a-tDCS served to ‘block’ certain higher-order cognitive performance gains on measures of inhibition, innovation, and memory for facts. Nonetheless, we did find evidence that participants, combined across groups, showed immediate (T2) cognitive gains in executive functions of conceptual reasoning and fluency (verbal and category fluency) as well as on a screening measure of memory (i.e., CVLT). On the latter screening measure (CVLT), both groups made comparable gains despite significantly different baseline means on CVLT. The combined groups also showed significant immediate (T2 to T1) and persisted (T3 to T1) improvement in subjective satisfaction in memory abilities [Multifactorial Memory questionnaire (MMQ)] reported via questionnaire.

To our knowledge, the present randomized pilot represents one of the first studies in MCI to compare a-tDCS directed toward left IFG versus s-tDCS, delivered before each cognitive training session. The contradictory findings to our expectations suggest that the anode and cathode placement sites we chose, i.e., left IFG and right arm respectively, failed to incrementally improve cognition above that achieved by SMART training alone, despite clear evidence that the tDCS altered rCBF. What is important to note is the higher rCBF at T2 (end of training period of eight sessions) was in the contralateral prefrontal cortex to the one stimulated, not the region beneath the stimulated left IFG.

The key question that emerges is why a-tDCS over left IFG ‘blocked’ rather than enhanced neuromodulation effects of cognitive training (SMART) on relevant cognitive measures in this MCI randomized pilot trial. We offer several possible explanations for our unexpected findings that should be explored in subsequent trials to better understand the underlying mechanisms contributing to or blocking additive benefits of tDCS to cognitive training. Transcranial direct current stimulation has shown to modulate cortical plasticity that can be manifested as either excitatory or inhibitory (Prehn and Flöel, 2015). A plausible explanation for why the a-tDCS did not enhance performance in this pilot trial could be that the direct current applied to left IFG may have modulated the resting membrane action potential (AP) inducing inhibitory hemostatic mechanism, and thereby reducing the neuroplasticity of subsequent learnings during cognitive training (Lang et al., 2003; Peters et al., 2013). A second alternative is that the multiple sessions of a-tDCS to left IFG perturbed the spontaneous firing rates of neural networks thereby blocking the consolidation of top-down learned strategies of SMART training (Peters et al., 2013). In order to explore the real-time neural changes induced by tDCS, future studies should evaluate neural response during stimulation with measures such as immediate changes in rCBF, alterations in resting-state functional connectivity or changes in EEG, to mention a few. Another possible explanation for the ‘blocked’ effects may be the a-tDCS triggered a reallocation of CBF to the contralateral side to the site stimulated, disrupting the underlying neural network subserving these higher-order cognitive abilities. Support for this possible explanation of the ‘blocking effects’ of the a-tDCS arises from prior evidence that disrupted right prefrontal cortical function interferes with holistic processing, such as that assessed by our innovation measure (Heilman et al., 2003; Siddiqui et al., 2008). Whether the detrimental impact of a-tDCS prior to cognitive training, implicated in this pilot project, is due to the fact that the brain was already compromised by MCI, or whether this would be the case for other populations, either healthy or those with more focal injuries, remains to be explored.

One pattern that remains equivocal is whether higher or lower rCBF represents positive or maladaptive neural changes (Chapman et al., 2016). Seemingly the increased rCBF was not linked to adaptive gains in the present study. However, it is important to point out that we did not find a relationship between lower cognitive performance and either higher versus lower rCBF in the present study, possibly due to small sample size. Therefore, we interpret our findings that the increased rCBF in the right MFC may be a maladaptive response to stimulation of the left frontal area cautiously. The present pilot findings raise more questions than it answers and points to the importance of seeking converging patterns between cognitive and neural changes resulting from intervention protocols, to better understand the neural mechanisms related to both positive and negative effects. Whereas we had anticipated the neural changes to be more closely identified in the site of stimulation, (i.e., left prefrontal cortex), other work has shown remote changes from the stimulation site. Similar to our findings of increased CBF in alternate brain regions from the one stimulated, Yun’s study (Yun et al., 2016) which measured glucose utilization a proxy for rCBF using resting 18-Fluorodeoxyglucose positron emission tomography (18FDG-PET) observed increased glucose uptake in non-stimulated area i.e., dorsolateral, ventrolateral, medial prefrontal cortices, the anterior and posterior insula, the hippocampal and parahippocampal areas, and the dorsal anterior cingulate but not in the stimulated region.

The present pilot trial provides data to glean insights to guide future endeavors which incorporate tDCS to test as a viable additive intervention option in MCI. The factors to consider that may help improve upon our current methodology include, but are not limited to: (1) the mechanism of action resulting from tDCS, (2) the electrode placement for stimulation (anodal and cathodal), (3) timing, length, and frequency of stimulation, (4) population under-study, and (5) measures adopted to examine effectiveness.

### Mechanism of Action

An important finding from our study was that a-tDCS directed over the left IFG was associated with increased rCBF to the right MFC. We cannot ascertain whether the tDCS stimulation effect alone created the changes in right frontal brain region or whether it was a combination effect of the training + tDCS effects. Prior work has shown that the ‘site of stimulation’ is the general area beneath the anode site, however, the neural alterations can occur across a larger neural network than the stimulated region (Yun et al., 2016). Our findings support the growing evidence that the tDCS impact is not directly related to the specific brain region under the electrode (Polanía et al., 2011). Our preliminary data suggest that the tDCS served to modulate neuronal function across specific brain networks since the left and right frontal regions are highly interconnected (Greicius et al., 2003). Thus as implicated above, the inhibitory effects from left frontal cortex may represent a generalized network effect that spread to the right frontal cortex.

Whereas the tDCS stimulation attenuated cognitive training effects on select measures, i.e., inhibition, innovation and complex fact memory (episodic memory) or episodic memory; other processes of complex abstraction and memory were less affected. Thus, it is important to point out that tDCS did not have a global inhibitory effect on all cognitive domains. Nonetheless, tDCS did not enhance any of the cognitive gains over and above the levels accelerated by training alone in the present study. We recognize that the pattern of findings is limited by our methodology and sample size.

### Montage Placement

The general anodal and cathodal placement that we utilized in the present study did not result in enhanced cognitive training effects. One next possible alternative methodology to test would be to stimulate the right frontal cortex to test whether this site of stimulation would increase rCBF to left frontal cortex. It is possible that stimulation to the contralateral frontal brain region may have enhanced, rather than attenuated, frontally mediated higher-order cognitive domains such as innovative thinking and complex inhibitory responses since prior evidence has supported left frontal cortex increased CBF associated with higher cognitive performance following training (Chapman and Mudar, 2014; Chapman et al., 2015). Additionally, the placement of the cathode electrode may impact the outcomes. A handful of other studies using tDCS in MCI applied the cathode stimulation to the right frontal polar cortex (Meinzer et al., 2015); whereas we chose to place the cathode on the right arm motivated by prior study in MCI and AD (Ferrucci et al., 2008; Boggio et al., 2012; Cotelli et al., 2014). The rationale for this cathode electrode placement was that we did not want to deactivate the opposite side of the brain region i.e., right frontal cortex, a hub for modulation of attention and creativity the summary of SMART training (Shruti et al., 2015).

### Timing and Frequency

Another major difference between our methodology and the one used by Meinzer et al. (2015) in MCI was they stimulated during active task performance rather than prior to learning new cognitive strategies, as in our study. We chose to stimulate prior to training based on prior evidence that tDCS can enhance the brain’s readiness to respond to subsequent learning (Prehn and Flöel, 2015). Subsequent trials should test whether anodal stimulation during training instead of just prior to training would enhance gains. We only provided stimulation and training twice a week. More intensive stimulation within shorter time intervals may be necessary to keep the brain primed to benefit from training.

### Population

Overall, the results support prior evidence (Mudar et al., 2017, 2019) that individuals with MCI can benefit from cognitive training, which in this case was SMART, as manifested by cognitive gains in the s-tDCS + SMART and both groups combined (sham + a-tDCS) with SMART. The current finding of generalizability of SMART to non-trained cognitive gains to other domains such as fluency and daily life function as measured by the MMQ (subjective memory evaluation) is promising. Additionally, the emergence of gains in the perceived facility in using memory strategies MMQ-S, although not observed immediately, but rather at 3 months after the training ended, may be due to a strengthening of self-confidence persisting from training. Previous work has shown that individuals manifest gains after SMART training has ended, hypothesized to emerge as individuals continue to utilize and habitually apply strategies learned during SMART in their daily life. Similar findings of SMART benefits were found across different studies in both healthy and clinical populations such as traumatic brain injury and bipolar disorders (Chapman et al., 2015; Vas et al., 2016; Venza et al., 2016). Not only does cognitive training strengthen cognitive and neural abilities but emerging evidence suggests that strategy-based cognitive training might be beneficial in mitigating dementia onset. The Advanced Cognitive Training for Independent and Vital Elderly (ACTIVE) RCT study in older cognitively normal adults, which implemented three cognitive training models targeting: memory, reasoning, and speed of processing, found that speed of processing training was able to decrease the rate of dementia by 29% over a 10 years’ period (Edward et al., 2017). Thus, our findings add to the prior knowledge that cognitive training can help strengthen cognitive functions not only in cognitively normal elderly but also in individuals with MCI and perhaps even slow rate of cognitive losses.

Moreover, cognitive gains, when both groups were combined, showed not only gains in individual’s subjective memory perception [Multifactorial-Memory Questionnaire(MMQ)] as noted above, but also on the objective measure of memory [California Verbal Learning Task (CVLT)], a test that was used to characterize MCI in participants as screening criteria. On this objective memory measure, the combined groups showed significantly higher scores immediately (T2) and over 3-months post-training (T3) period. Taken together, these results suggest that SMART impacted not only enhanced higher-order cognitive performance but also showed generalized effects on memory, both self-reported (MMQ) and measured (CVLT).

The failure to find significant group differences on the subjective and objective memory measures may be due to the small sample size as we were moderately powered for assessing the interaction effects, though well-powered for all neurocognitive tests assessing the SMART effect alone. The limitation of low power of the study could explain the discrepancy of our study findings from Yun’s study (Yun et al., 2016) which showed substantial improvement in subjective memory rating (MMQ) only in a-tDCS group compared to the s- tDCS stimulation over left DLPFC. One more issue of consideration is where the individuals are along the MCI to Alzheimer’s dementia continuum in terms of disease severity. For example, based on Cotelli’s work (Cotelli et al., 2014) in 36 AD patients failed to show significant cognitive gains when computerized memory training was combined with a-tDCS stimulation to the left DLPFC with a constant current of 2 mA for a total of 10 sessions for 25 min per session over 2 weeks compared to sham group. Perhaps our participants were more impaired than those in Yun’s study.

### Measures

The majority of tDCS studies have investigated the benefit on primary motor cortex and isolated cognitive processes. Our results suggest that tDCS may have limited beneficial adjuvant effects in recruiting cortical plasticity to enhance higher-order cognitive processes in individuals who are at a greater risk of cognitive decline. In the current study, a-tDCS over left IFG served to attenuate gains of SMART on complex measures of inhibition, innovative cognition as measured by fluency of multiple interpretations, and episodic memory as measured by recall of facts from complex textual information. Nonetheless, even in a healthy aging group, the result supports that consolidation of visual learning could be blocked as soon as second day, using a-tDCS stimulation for 20 min and 2 mA current on primary visual cortex (Peters et al., 2013). In line with our work and similar other work on motor cortex and visual learning (Lang et al., 2003; Peters et al., 2013), one presumable hypothesis is that repeated stimulation to vulnerable region i.e., IFG blocked the consolidation of frontally mediated top–down learning strategies central to SMART training. Moreover, it is unclear to what extent these present findings are transferable to other methodological manipulations such as loci of stimulation and stimulation during or prior to trainings.

## Conclusion

This study provides evidence that a-tDCS to the left frontal cortex does affect cognitive-neural changes in MCI, but not in a direction that supported a view that tDCS presented just prior to cognitive training elevated higher-order cognitive training benefits or served to ameliorate cognitive dysfunction. That is, the loci and timing of stimulation adopted in the present study did not replicate prior findings suggesting a potential modifying effect of tDCS on MCI (Meinzer et al., 2015; Yun et al., 2016; Murugaraja et al., 2017; Gonzalez et al., 2018). Future work is needed to examine precisely what happens to brain when tDCS is applied using fMRI studies to better understand the action of tDCS at the neuronal level. Strengths of the study are the randomized control design, the inclusion of broad-based cognitive measures, well-defined MCI population based on widely accepted criteria, and replication of prior findings that individuals with MCI can benefit from top–down cognitive training, namely SMART. Prior evidence has shown consistent data that cognitive training may be one of the most promising currently available interventions to impact disease progression (Edward et al., 2017).

Moreover, the most important contribution of the present study is that it adds to the growing body of compelling evidence that cognitive training provides an intervention option to benefit people today. Instead of feeling no options exist, this work supports prior evidence that being proactive about cognitive brain health may reap benefits in strengthening cognitive capabilities. Further studies are required to explore the short-term and long-term cognitive and neural benefits of combined non-pharmacological interventions in MCI with the goal to develop protocols that delay progression of MCI to AD. Indeed, keeping the mind stimulated may be a key aspect to mitigating some age-related aspects of cognitive decline or MCI-specified deficits. This study motivates future work to test potential additive effects of tDCS stimulation during training and potentially different loci of stimulation. Nonetheless, this initial pilot supports the view that tDCS stimulation techniques are safe to apply to the human brain and do cross the bony protection of the skull into effect brain change.

## Author Contributions

ND has been the key player in the research, analysis of the data, and writing the complete manuscript. SC was the principal investigator of the study and has been an integral part in the completion of the manuscript. JS played a key role in analyzing the data. SA was involved in MRI analysis. SV and AR were an integral part in the development of tDCS research protocol and implementation of the research. AR involved in the recruitment of the participant. RM helped in reviewing the article and give valuable feedback. MQ is board certified neurologist that confirmed the diagnosis of MCI in the study.

## Conflict of Interest Statement

The authors declare that the research was conducted in the absence of any commercial or financial relationships that could be construed as a potential conflict of interest.
